# Dopaminergic dysregulation syndrome associated with Othello’s syndrome in a patient with Parkinson’s disease: about a clinical case

**DOI:** 10.1192/j.eurpsy.2022.1195

**Published:** 2022-09-01

**Authors:** R. Masmoudi, F. Cherif, S. Hentati, R. Sallemi, I. Feki, J. Masmoudi

**Affiliations:** 1 Hospital university of HEDI CHAKER, Psychiatry A Department, Sfax, Tunisia; 2 HEDI CHAKER hospital, Psychiatry A Department, SFAX, Tunisia; 3 CHU Hedi CHaker hospital Sfax Tunisia, Department Of Psychiatry (a), Sfax, Tunisia

**Keywords:** Dopaminergic dysregulation syndrome, Parkinson, Othello syndrome

## Abstract

**Introduction:**

Parkinson’s disease (PD) and its pharmacological treatment can be associated with a long list of neuropsychiatric complications.

**Objectives:**

The aim of our case report is to investigate through a case analysis the possible association of dopaminergic dysregulation syndrome and Othello syndrome

**Methods:**

we carried out a case analysis and a review of the literature by searching the PubMed database

**Results:**

Case report We present the case of a 43-year-old man suffering from early PD since the age of 16, started on levodopa since the age of 19.

Since 2 years, the patient has resorted to a considerable increase in the doses of levodopa up to 2500 mg / day, the evolution was marked by the installation of disabling dyskinesias and by a change in his behavior and mood.

He was then hospitalized in psychiatry following aggressive behavior towards his wife. The admission examination found a patient who was motor unstable with an interpretative delirium of jealousy and persecution.

The diagnosis of Othello syndrome associated with SDD was retained.

Our therapeutic strategy has been to put the patient on quetiapine, reduce the doses of levodopa, add a dopamine agonist and involve psychoeducation of the patient and his family. Evolution has been marked by the reduction of delusions of jealousy.

**Conclusions:**

This case reports a rare case of delirium of jealousy induced by the misuse of dopaminergic drugs in a patient with PD in its early form. These complications can have catastrophic consequences for the patient. Researching and recognizing these psychiatric manifestations should help avoid devastating consequences.

**Disclosure:**

No significant relationships.

